# Recurrent primary mediastinal giant cell tumor of soft tissue with radiological findings: a rare case report and literature review

**DOI:** 10.1186/s12957-017-1205-5

**Published:** 2017-07-26

**Authors:** Qiongjie Hu, Jian Peng, Liming Xia

**Affiliations:** Department of Radiology, Tongji Hospital, Tongji Medical College, Huazhong University of Science and Technology, Jiefang Dadao 1095(#), 430030 Wuhan, People’s Republic of China

**Keywords:** Giant cell tumor, Soft tissue, Mediastinum, Recurrence, Imaging, Magnetic resonance imaging

## Abstract

**Background:**

Giant cell tumor of soft tissue (GCT-ST), which histologically resembles GCT of bone, is a rare tumor. Usually, it is located in the lower extremities and trunk. GCT-ST, occurring in mediastinum, is extremely rare.

**Case presentation:**

We encountered an 18-year-old Chinese woman who had mild dull pain on the left side of back. The following chest computed tomography (CT) showed a heterogeneous mass deeply situated in the posterior mediastinum with compression of the lung and invasion of the adjacent rib. On magnetic resonance imaging (MRI), the tumor exhibited predominantly slight hyperintensity on T2-weighted images and intensely heterogeneous enhancement on contrast-enhanced T1-weighted images. The whole body bone scan showed a mildly increased radiotracer uptake in the proximal portion of the left fifth rib, suggestive of local infiltration by the tumor. Surgical resection of the tumor was performed; subsequently, the tumor was histopathologically proved as GCT-ST. Three months after the operation, the patient developed a local recurrence. A brief discussion about the radiological findings, histopathological features, clinical behavior, and a detailed review of the relevant literature are presented.

**Conclusions:**

To the best of our knowledge, this is the first case about recurrent primary mediastinal GCT-ST, moreover, this is the first report to introduce the MRI findings of primary mediastinal GCT-ST. The present case highlights the ubiquitous distribution of soft tissue giant cell tumor and the importance of considering this tumor in the differential diagnosis of posterior mediastinal neoplasms. Also, a long-term follow-up is required to properly assess the malignant potential of this tumor.

## Background

Giant cell tumor of soft tissue (GCT-ST) is a rare tumor, which was first described by Salm and Sissons in 1972 as a distinct entity [1]. GCT-ST resembles osseous giant cell tumor (GCT), which demonstrates a spectrum of benign to malignant characteristics [2]. This tumor, which has been described in numerous anatomic sites including the extremities, trunk, head and neck, tendon sheaths, skeletal muscle and skin can occur in both superficial and deep soft tissues [3]. Involvement of mediastinum by GCT-ST is even rarer. Only four cases of primary mediastinal GCT-ST have been reported in literature, and none of these cases show evidences of recurrence [4–6]. We herein describe an interesting case of a posterior mediastinal GCT-ST with postoperatively local recurrence, which was identified on plain films, CT, MRI, and single photon emission computed tomography (SPECT). Moreover, to our knowledge, there has been no report introducing the MR findings of primary mediastinal GCT-ST before.

## Case presentation

In April 2016, an 18-year-old female came to the outpatient clinic with a 3-week history of mild dull pain on the left side of back, which was not relieved after taking Nimesulide. There was no fever, cough, chest pain, hemoptysis, and dyspnea accompanying with the pain. The patient denied any history of smoking, alcohol consumption, exposure to tuberculosis, or tumor. There was no weight loss since onset. Her family history was unremarkable. No positive findings were obtained in the chest exam. Thus, the patient was sent for medical imaging examinations.

**Fig. 1:**
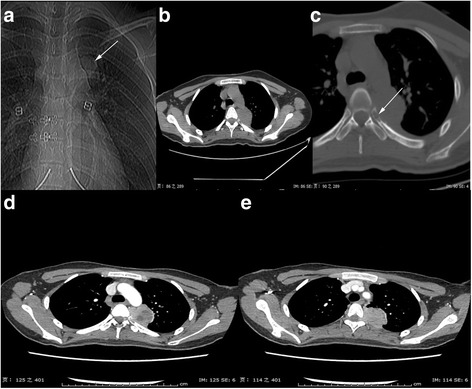
**a** X-ray revealed a mass located in the left mediastinal. Non-contrast **b**, **c** and contrast-enhanced **d**, **e** chest CT showed a heterogeneous mass deeply situating in the posterior mediastinum and enhancing inhomogeneously. The mass showed an obvious extension through the intervertebral foramen. **d** Compression of the lung and invasion of the adjacent rib are visualized

Chest X-ray showed a well-demarcated left mediastinal mass (Fig. 1a). Non-contrast chest CT (Fig. 1b, c) revealed a well-defined heterogeneous mass (measuring 39 mm × 35 mm × 41 mm) deeply located in the posterior mediastinum, extending from thoracic vertebrae D4 to D5. Compression of the lung and destruction of the posterior end of the left fifth rib were visualized (Fig. 1c). On the contrast-enhanced CT, the tumor manifested obviously inhomogeneous enhancement (Fig. 1d, e), furthermore, extension of the tumor through the intervertebral foramen into the spinal canal was distinctly displayed (Fig. 1e). Mild pleural effusion was also observed in the left pleural cavity.

**Fig. 2:**
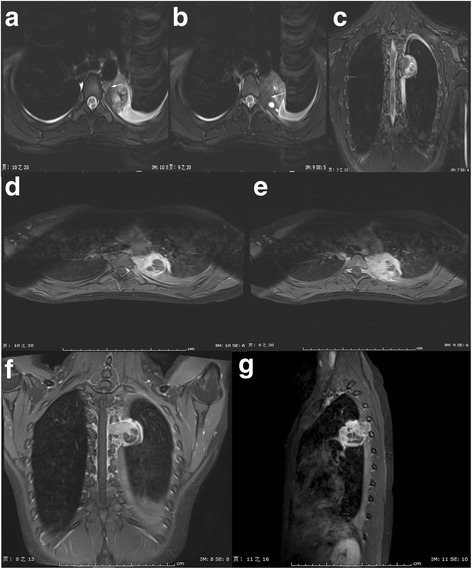
**a–c** Axial and coronal T2-weighted images showed a heterogeneous mass, whose main component was solid, mixed with small portion of cysts with different sizes. Moreover, inside the solid portion of the tumor, tiny septae of hypointensity (*white arrow*) were detected. **d–g** Gd-enhanced T1-weighted images showed intensely heterogeneous enhancement. The solid portion with septae inside and the walls and septae of the cysts displayed a significant enhancement

MRI provided more information on the observation of the inner components of the tumor due to the higher soft tissue resolution compared to CT. On the T2-weighted images (TR/TE, 4300/97 ms) (Fig. 2a–c), the tumor appeared a heterogeneous mass, whose main component was solid, mixed with small portion of cysts with different sizes. The solid portion of the mass appeared isointensity as the muscle, the cysts showed hyperintensity just as the cerebrospinal fluid, the walls of the cysts and the septae inside the cysts showed hypointensity as the muscle tendon. Moreover, within the solid portion of the tumor, tiny septae with hypointensity were observed. These septae divided the solid portion of the tumor into small parts with different sizes. On the Gd-enhanced T1-weighted images with fat suppression (TR/TE, 500/10 ms) (Fig. 2d–g), the solid portion with septae inside and the walls and septae of the cysts displayed a remarkable enhancement, while the cystic fluid showed no enhancement. MRI also clearly showed the extension into intervertebral foramen and the invasion of the adjacent rib, just like the previous contrast-enhanced CT did.

To evaluate if there was any other bone involved, SPECT was carried out. The whole-body bone scan (Tc-99 m-MDP bone scintigraphy) was suggestive of a local invasion in the posterior end of the left fifth rib by the tumor, while other bones appeared normal.

Serum calcium, phosphate, and alkaline phosphatase were within normal limits. Serum neuron-specific enolase (NSE), cytokeratin 19 fragment antigen 21-1 (CYFRA21-1), squamous cell carcinoma antigen (SCCAg), carcino-embryonic antigen (CEA), and β-human chorionic gonadotrophin (β-HCG) levels were all not elevated.

**Fig. 3:**
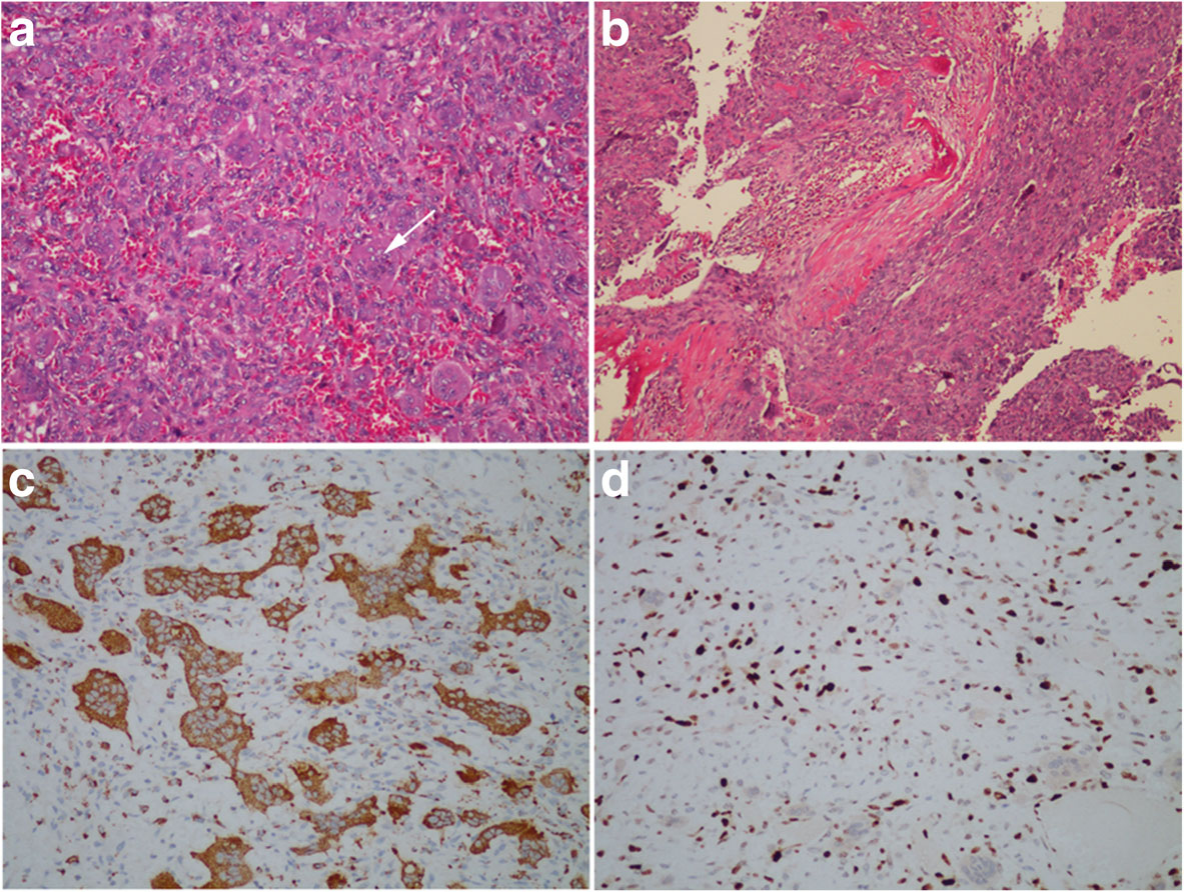
HE. **a** Mononuclear cells and multinucleated osteoclast-like giant cells were showed (×200). **b** Blood-filled cystic spaces were showed (×100). **c**, **d** Immunohistochemical staining (×200). CD68 showed cytoplasmic positivity. KI-67 labeling index was high

A thoracoscopic tumor resection was performed. The mass was approximately 50 mm × 50 mm × 50 mm in size, highly vascular, focally hemorrhagic, and adherent to the aorta and the upper lobe of left lung. There was no pleural effusion, metastasis, or atelectasis. After careful separation, the tumor was completely removed, and the lesion of the involved rib was also removed. Histological examination disclosed a lot of blood-filled cavities similar to those present in aneurysmal bone cyst, and a mixture of mononuclear cells and multinucleated osteoclast-like giant cells with high mitotic activity (12 figures per 10 HPFs) and no notable nuclear atypia (Fig. 3a, b). Immunohistochemically, the tumor showed strong positive reaction in the giant cells to the histiocytic marker CD68 (Fig. 3c) and positive reaction in the mononuclear component to antibodies against vimentin and CD163. Ki-67 labeling index ranged from 25 to 30%, indicating nuclear positivity (Fig. 3d). Above all, the final pathological diagnosis was mediastinal GCT-ST of low malignant potential.

**Fig. 4:**
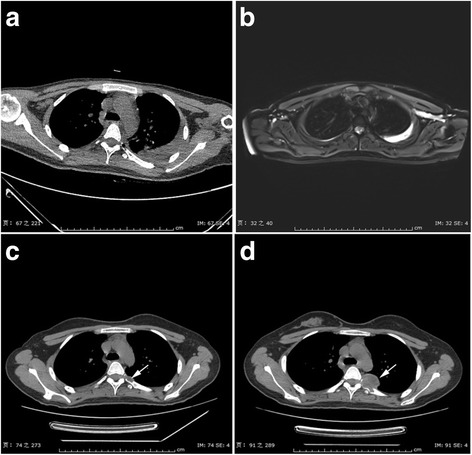
**a** 5 days after operation. **b** 1 month after operation. **c** 3 months after operation. **d** 5 months after operation. The local recurrence of the tumor was demonstrated 3 months after operation and developed much bigger 2 months later

The patient reviewed three times after her operation. The first follow-up was 1 month after operation, when a MRI scan (Fig. 4b) was performed, no signs of tumor recurrence were found. Two months after the first review, the patient came for the second review with a complaint of mild dull pain on the left side of back, then a CT scan was performed (because of financial problem, she refused MRI examination), which detected a small soft tissue nodule (measuring 13 mm × 8 mm × 10 mm) right in the operative region of the posterior end of the left fifth rib (Fig. 4c). The patient left without any treatment after being told the situation of tumor recurrence. The third follow-up was another 2 months after the second review, which showed the recurrent tumor becoming much bigger (measuring 34 mm × 28 mm × 31 mm) (Fig. 4d) than found in the second review.

## Discussion

Giant cell tumor of soft tissue (GCT-ST) is a rare tumor. GCT-ST, which resembles osseous GCT [2], broadly encompasses GCT of soft tissue without any osseous origin. It happens in any age, but usually it is found in middle-aged adult without gender difference [2, 3]. The patients often have no symptoms or only have mild pain. Notice of a painless growing mass is the most common complaint [2, 3]. The tumor occurs in both superficial and deep soft tissues [2, 3]. The lower limbs, particularly the thighs [2, 4] are the most frequent locations. Other sites of occurrence include the trunk, the upper extremities, and rarely the abdomen and skin [3, 4]. Involvement of mediastinum is extremely rare. In this report, we provide important information regarding diagnostic imaging (plain film, CT, MRI, and SPECT) from a case of recurrent GCT-ST of low malignant potential at the very rare site of mediastinum.

To the best of our knowledge, only four cases of primary mediastinal GCT-ST have been reported in the literature [4–6]. For these 5 cases of mediastinal GCT-ST, the ages ranged from 18 to 53 years, with 2 males and 3 females. The sizes of the tumors ranged from 2.5 to 15 cm. Two of the cases had no bone involvement, while three had adjacent bone invasion. Microscopic examination demonstrated that all the tumors were composed of a mixture of osteoclast-like giant cells and mononuclear cells with a background of high-vascularity stroma. Cystic change and hemorrhage were seen in three cases. Mitotic rates were high (20 to 30%) in two cases, whereas they were very low (less than 5%) in the other three cases. All the patients had undergone surgical resections of the tumor and follow-up. The previous four cases presented no tumor recurrence during the follow-up, whereas the patient in our case developed a local recurrence 3 months after the operation. Folpe et al. [7] studied 31 cases of GCT-ST and proposed the term “giant cell tumor of low malignant potential” due to the features of mild or moderate atypia of the tumor. Additionally, O’Connell et al. [3] described a series of 18 GCT-ST patients which demonstrated a spectrum from benign to malignant behavior.

Medical imaging examination plays an important role in locating the exact origin and observing the concrete details of the tumor. Fu et al. [4] reported chest X-ray manifestations of two GCT-ST cases. In the present case, comprehensive medical imaging examinations (plain film, CT, MRI, and SPECT) were carried out. Chest film showed a mass with clear and smooth boundary at the left side of mediastinum, just similar to the previous reports [4–6]. Chest CT showed the mass situating deeply in the posterior mediastinum. The tumor appeared heterogeneous hypo-attenuation (compared to chest muscles) on pre-contrast CT and inhomogeneous enhancement on post-contrast CT, which were consistent with the early reports [5, 6]. Invasion of an adjacent rib was also detected in our case, similar to that reported by Goldberg et al. [5] and Jain et al. [6]. Interestingly, our case showed an extension of the tumor through the intervertebral foramen into the spinal canal, which had never been reported in literature. MRI has a much higher resolution in soft tissue imaging than CT. However, to our knowledge, there has been no report introducing the MR findings of primary mediastinal GCT-ST before. The first time we read the MRI images of the patient, we only noticed the gross appearances of the tumor: paravertebral mass, patchy liquid signal inside, significantly inhomogeneous enhancement, and the extension through the intervertebral foramen, just the same as what we found from the CT images. In our daily work, the most common tumors in the posterior mediastinum are lymphoma, metastatic tumor, and schwannoma. Lymphoma usually displays as multiple moderately enhanced homogeneous nodules or masses with no adjacent bone destruction. Metastatic tumor must be secondary to primary tumor, while medical history, physical examination, and blood tumor markers of the patient showed no evidence of primary tumor. The schwannoma shows an inhomogeneously enhanced mass with necrosis inside, and it grows along the nerve sheath and sometimes extends into the intervertebral foramen. Thus, we made the initial diagnosis of schwannoma. After the pathological results came out, we analyzed the MRI images retrospectively, trying to find some imaging features that contribute to the diagnosis of this rare tumor. GCT-ST is a rare neoplasm, histologically resembling GCT of bone [8–10]. According to the histological features of GCT-ST [11–13], the tumor shows multi-nodular structure under low power field, which varies in size and separated by fibrous connective tissue. Some of the nodules can appear cystic change, while necrosis of the tumor cell is hardly to see. On T2-weighted images, tiny septae showing hypointensity were detected inside the solid portion of the tumor and the cysts, which represented the fibrous connective tissue. Patchy liquid signals were misunderstood as necrosis when we first read the images, as we ignored the hypointensity walls surrounding them. In fact, other tumors, such as liver cancer and schwannoma, do not show a hypointensity wall around the necrosis zone. Here in this tumor, the thin walls surrounding the liquid signals indicated that these patchy liquid signals were actually the cystic change of the tumor nodules rather than necrosis.

GCT-ST is rare, the pathogenesis of this tumor is still unclear, and the effective adjuvant therapy after operation is also under exploration. It is desirable to acquire further accumulation of more cases accompanied with medical imaging findings, histopathological features, and cytogenetic and molecular pathological analyses.

## Conclusions

In conclusion, our case illustrates a primary GCT-ST located in the posterior mediastinum with post-operative recurrence. Pre-operative diagnosis of primary mediastinal GCT-ST is challenging, diagnostic imaging plays a very important role in obtaining the comprehensive information of the tumor, including the location, size, internal condition, growth pattern, and the relationship with adjacent tissues, to achieve the diagnosis. For tumors which are located in the posterior mediastinum with aforementioned MRI findings, the diagnosis of GCT-ST should be considered. However, since the GCT-ST is a rare tumor, its imaging characteristics, especially the MRI characteristics, need to be summarized by collecting more cases in the future, and the final diagnosis depends on the histopathological examination. Postoperatively, close follow-up is necessary, because of the tumor’s malignant potential which may lead to a recurrence.

## Abbreviations

CEA: Carcino-embryonic antigen
CT: Computed tomography
CYFRA21-1: Cytokeratin 19 fragment antigen 21-1
GCT: Osseous giant cell tumor
GCT-ST: Giant cell tumor of soft tissue
MRI: Magnetic resonance imaging
NSE: Neuron-specific enolase
SCCAg: Squamous cell carcinoma antigen
SPECT: Single photon emission computed tomography
β-HCG: β-human chorionic gonadotrophin
